# Geographic Rounding on Young Adults With Childhood-Onset Chronic Conditions: A Mixed Methods Retrospective Observational Cohort Study

**DOI:** 10.7759/cureus.110993

**Published:** 2026-06-16

**Authors:** Samantha H Collins, Alyssa Platt, Jesse Rhodes, Tyler White, Maragatha Kuchibhatla, Ruchi Doshi

**Affiliations:** 1 Departments of Internal Medicine and Pediatrics, Duke University School of Medicine, Durham, USA; 2 Department of Hematology and Oncology, Columbia University, New York, USA; 3 Department of Biostatistics and Bioinformatics, Duke University School of Medicine, Durham, USA; 4 Performance Services, Duke University Health System, Durham, USA

**Keywords:** 30-day readmission, adolescent young adult populations, bedside rounding, chronic disease, length of hospital stay, nursing workload (nwl)

## Abstract

Background

Geographic rounding has been associated with improved efficiency and quality of care on inpatient general medicine services. Young adults with childhood-onset chronic conditions (COCC) often experience longer lengths of stay and higher readmission rates than other general medicine populations. To evaluate whether geographic rounding could improve outcomes for this group, these patients were transitioned to a geographic team.

Methods

We conducted a retrospective observational cohort study comparing length of stay (LOS) and 30-day readmission rates for hospitalized young adults with COCC before and during geographic rounding. Nursing workforce data and workload during the geographic period were analyzed, using a comparable non-specialty geographic unit as a reference. A limited qualitative analysis of a nursing survey was performed to assess attitudes and experiences related to caring for this specialty population.

Results

LOS was significantly longer during the geographic period (event time ratio=1.29, 95% CI: 1.04 - 1.57). There was no statistically significant difference in readmission rates (risk ratio: 1.45, 95% confidence interval (CI): 0.85 - 2.41). Although measured nursing workload on the geographic unit was similar to that of other geographic units, nurses reported substantial burnout, challenging patient dynamics, and inadequate resources to meet the complexity of patient needs.

Conclusions

In this specialty population of young adults with COCC, geographic rounding was associated with worse efficiency and was accompanied by unintended workforce challenges. These findings suggest that geographic rounding may not be universally effective for medically and socially complex populations. Implementation of geographic models for specialty cohorts should include early partnership with nursing staff and appropriate training and resource allocation to support both patient care and workforce sustainability.

## Introduction

Geographic rounding refers to the cohorting of hospitalized patients on the same physical hospital unit with a limited number of provider teams and consistent clinical staff. In general medicine populations, geographic rounding has been associated with improved care quality [1], educational experience for medical trainees [2], and operational efficiency [2,3]. Prior studies have also described improvements in nursing and provider communication and interdisciplinary teamwork [1-4], particularly when geographic rounding is paired with multidisciplinary rounds (MDRs) [5,6]. In these settings, geographic rounding with MDRs has been associated with reduced length of stay (LOS) [5,6] and 30‑day readmission rates [5].

Young adults with childhood‑onset chronic conditions (COCCs) represent a distinct inpatient population with complex medical, psychosocial, and transitional care needs. Compared to the broader general medicine population, these patients historically experience longer LOS and higher 30‑day readmission rates [7,8]. Dedicated Internal Medicine‑Pediatrics (MP) service lines designed for young adults with COCCs (referred to as MP-COCC) [9] have been associated with lower readmission rates, although these improvements may be accompanied by longer hospital stays [8].

Despite evidence supporting geographic rounding in general medicine populations [1-6], its effectiveness in specialty cohorts such as young adults with COCCs remains unclear. Further, there exists limited data on the experience of nursing with certain patient cohorts concentrated on single units. Given prior success of geographic rounding combined with MDRs, geographic rounding was started on the MP‑COCC service line with the hypothesis that this implementation would improve LOS, readmission rates, and care quality. This retrospective observational cohort study leverages both quantitative and qualitative methods to investigate our primary objective to evaluate the impact of geographic rounding on LOS and 30‑day readmissions for hospitalized young adults with COCCs, and our secondary objective, to examine nursing workload, workforce outcomes, and perceptions of providing geographically based care for this population.

## Materials and methods

Study design

We conducted a retrospective observational cohort study using patient-level electronic health record (EHR) data combined with nursing workforce, workload, and survey data to evaluate implementation of geographic rounding for young adults with COCCs. The study population included adults (≥18 years) identified as MP-COCC patients hospitalized on the MP-COCC service line at a single academic medical center between January 2022 and October 2023, spanning periods before and after geographic rounding was implemented.

We integrated comparative analyses of EHR-derived patient outcomes with descriptive analyses of nursing workforce and workload data and qualitative assessment of nurse survey responses to contextualize observed outcomes. The Institutional Review Board approved this study (approval number: Pro00104906) and determined it exempt from full review as a quality improvement initiative. Patients and the public were not involved in the design or development of this study.

Geographic rounding

Population

Eligible encounters included adult patients hospitalized between January and October 2022 (non‑geographic period) or between January and October 2023 (geographic period) who were enrolled in the MP‑COCC service line (identified by MP physicians) and discharged by the MP‑COCC team during their inpatient stay. 

During the geographic period, encounters were included only if the patient was discharged from the geographic study unit (GSU) to ensure fidelity of exposure to geographic rounding. Our health system employs a team-based assignment model to facilitate geographic rounding at the system level. During the intervention period, patients assigned to the MP-COCC team admitted through the emergency department were preferentially placed on the GSU as beds became available. Patients requiring specialized nursing capabilities not available on the GSU were assigned to alternative units with appropriate resources. These patients, patients boarding in the emergency department at time of discharge, or patients admitted through the transfer center and not housed on the GSU, were not included in the study. Encounters outside the defined study periods were also excluded.

Intervention and Control

During the intervention period, MP‑COCC patients were assigned rooms on the GSU, resulting in consistent nursing staff and allied health team members (physical and occupational therapy, nutrition, pharmacy) in addition to the established MP‑COCC care team (hospitalist, licensed clinical social worker, and case manager) [9]. On the GSU, there were twice‑daily MDRs including nursing, therapy services, and the MP‑COCC team.

Prior to geographic rounding, MP‑COCC patients received care under a non‑geographic model in which the MP‑COCC team followed patients across multiple units throughout the hospital. This model resulted in variable nursing and allied health staffing and required coordination across multiple physical locations.

Data

Encounter‑level data were extracted from institutional EHR databases and included patient demographics, clinical characteristics, and hospitalization details. Each patient was assigned a dominant COCC following a structured chart review by a single investigator. Patients admitted for an exacerbation of a COCC (for example, vaso-occlusive crisis in an individual with sickle cell disease or ketoacidosis in an individual with type 1 diabetes) were assigned the exacerbated COCC. For patients who carried a higher level of medical complexity, three investigators reviewed the patient’s chart and came to a consensus on the dominant COCC driving their placement on the MP-COCC service. The datasets analyzed during the current study are available from the corresponding author on reasonable request.

Outcomes

The primary patient outcome was hospital LOS, measured in days from admission to discharge. The secondary outcome was 30‑day hospital readmission, defined as any unplanned readmission to a hospital within the same health system within 30 days of discharge [10].

Statistical Analysis

Patient sociodemographic and clinical characteristics were summarized using means with standard deviations or medians with interquartile ranges for continuous variables and frequencies for categorical variables. Overlap weighting [11] was used to balance characteristics between pre‑geographic and geographic MP‑COCC encounters. Covariates included age, gender, race, insurance type, residential location, admission type, intensive care unit stay, COCC category, and prior hospitalizations in the last 12 months. Distributions of propensity scores and pre- and post-weighting standardized differences were compared between geographic MP-COCC patient encounters and non-geographic MP-COCC encounters (with standardized differences < |0.10| considered acceptable balance) to assess differences in patient characteristics.

LOS was analyzed using accelerated failure time models [12] with a log‑logistic distribution, with inpatient death treated as a censoring event. Results are reported as event time ratios. Thirty‑day readmission was analyzed using Cox proportional hazards models, with death treated as a censoring event; proportional hazards assumptions were assessed using Schoenfeld residuals. For comparability, risk ratios were also estimated using log‑binomial regression.

All regression models included an indicator for geographic period as the primary exposure. Bootstrapping with 1,000 resamples at the patient level was used to estimate 95% confidence intervals and account for repeated measures and propensity score uncertainty. Analyses were performed using Stata version 19 (StataCorp, College Station, TX, USA) [13].

Nursing response to geography

Nursing-related outcomes during the geographic period included measures of workforce stability, workload intensity, and qualitative experiences of caring for the MP‑COCC population. For nursing workforce and workload analyses, the geographic comparison unit (GCU), defined as a general medicine unit without a specialty patient focus and with demonstrated prior benefit from geographic rounding and multidisciplinary rounds, served as a contextual comparison unit during the intervention period only.

Nursing Workforce

Nursing workforce data included average staffing levels and net monthly staffing changes within each unit derived from institutional human resources data. These data were obtained for both the GSU and the GCU.

Nursing Workload

The Nursing Workload Tool is a proprietary EHR‑based system that calculates a score reflecting the level of effort of care needed for a patient based on data pulls on recent documentation, active orders, and anticipated care tasks. For this analysis, unit‑level scores were created via aggregation of individual hourly patient scores over 24 hours and assigned a unit-level max, median, mean and standard deviation for each day within each unit, along with daily patient counts per unit.

Nursing workload data were extracted daily from the EHR for all adult patients receiving care on the GSU and GCU during January to October 2023. Workload scores were aggregated from patient‑level to unit‑level measures and summarized daily. No exclusions were applied. Workforce and workload analyses were exploratory and descriptive, intended to contextualize patient‑level outcomes rather than to assess causal relationships. Descriptive analyses of nursing workforce and workload trends are presented using run charts and smoothing plots.

Nursing Response to MP-COCC

In January 2024, nursing staff who regularly staffed the GSU were invited to complete an anonymous, three‑question free‑response survey assessing attitudes and experiences related to caring for MP‑COCC patients. There were no exclusion criteria. The specific questions (adapted for clarity) were: (A) What is going well with having MP-COCC patients geographically located on the GSU; (B) What is NOT going well with having MP-COCC patients on the GSU; and (C) Please share your honest feedback on caring for MP-COCC patients. 

Free‑response nursing survey data were analyzed using the framework method [14]. Three reviewers independently coded responses using a combination of deductive and inductive approaches and reached consensus on finalized themes. Themes were summarized by frequency at both respondent and code levels. Thematic saturation was reached, with no additions to themes in the last several survey responses. Qualitative findings were used to contextualize quantitative results. Qualitative themes are summarized using categorical visualizations.

## Results

**Figure 1:**
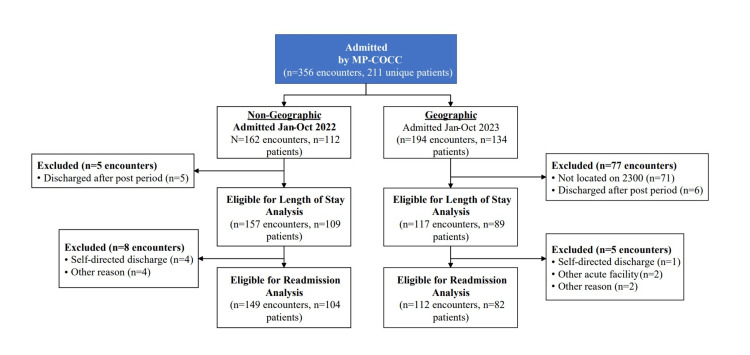
Strengthening the Reporting of Observational studies in Epidemiology (STROBE) diagram for patient flow from eligibility to analytic sample MP-COCC: Internal Medicine-Pediatrics childhood-onset chronic conditions

During the study period, 356 MP‑COCC hospital encounters were recorded, representing 211 unique patients across the pre‑geographic (January-October 2022) and geographic (January-October 2023) periods. Of these encounters, 162 occurred during the pre‑geographic period and 194 during the geographic period (Figure 1). Eighty-two encounters were excluded due to patients not being located on the GSU or for having discharge dates after the end of the study period. This left 274 encounters eligible to be analyzed for LOS and 261 eligible for analysis of 30-day readmission.

Across all eligible encounters, the mean patient age was 27.9 years (SD 7.1), with over one‑third of patients aged 30 years or older (Table 1). Sex distribution was balanced, and the majority of patients identified as Black or African American (54.4%). Most patients resided close to the hospital in the same or an adjacent county and were publicly insured. Sickle cell disease and diabetes mellitus were the most common childhood‑onset chronic conditions. At baseline there were high rates of readmission, with the majority of patients having been discharged at least once prior to the encounter. Baseline sociodemographic and clinical characteristics were similar across the pre‑geographic and geographic periods when assessed at the encounter level.

**Table 1:** Patient (encounter-level) characteristics before and after geographic rounding EDS: Ehlers-Danlos syndrome; POTS: postural orthostatic tachycardia syndrome

	Non-geographic	Geographic	Total
N	157 (57.3%)	117 (42.7%)	274 (100.0%)
Age (years)	27.5 (6.7)	28.4 (7.6)	27.9 (7.1)
Age (years)			
18-22	51 (32.5%)	32 (27.4%)	83 (30.3%)
23-29	54 (34.4%)	40 (34.2%)	94 (34.3%)
30+	52 (33.1%)	45 (38.5%)	97 (35.4%)
Gender			
Female	81 (51.6%)	61 (52.1%)	142 (51.8%)
Male	76 (48.4%)	56 (47.9%)	132 (48.2%)
Race			
White	58 (36.9%)	46 (39.3%)	104 (38.0%)
Black or African American	88 (56.1%)	61 (52.1%)	149 (54.4%)
Other	10 (6.4%)	10 (8.5%)	20 (7.3%)
Not Reported/Declined	1 (0.6%)	0 (0.0%)	1 (0.4%)
Ethnicity			
Not Hispanic/Latino	147 (93.6%)	114 (97.4%)	261 (95.3%)
Hispanic/Latino	7 (4.5%)	3 (2.6%)	10 (3.6%)
Not Reported/Declined	3 (1.9%)	0 (0.0%)	3 (1.1%)
Residential Location			
Hospital County	57 (36.3%)	31 (26.5%)	88 (32.1%)
Adjacent to Hospital County	44 (28.0%)	30 (25.6%)	74 (27.0%)
North Carolina	49 (31.2%)	43 (36.8%)	92 (33.6%)
Out-of-State	7 (4.5%)	13 (11.1%)	20 (7.3%)
Insurance type			
Managed Care	11 (7.0%)	4 (3.4%)	15 (5.5%)
Private	54 (34.4%)	39 (33.3%)	93 (33.9%)
Public	85 (54.1%)	69 (59.0%)	154 (56.2%)
Self-Pay	7 (4.5%)	5 (4.3%)	12 (4.4%)
Has PCP			
No PCP	45 (28.7%)	32 (27.4%)	77 (28.1%)
Has PCP	112 (71.3%)	85 (72.6%)	197 (71.9%)
Discharges in the last 12 months			
None	44 (28.0%)	34 (29.1%)	78 (28.5%)
1-3	62 (39.5%)	49 (41.9%)	111 (40.5%)
4+	51 (32.5%)	34 (29.1%)	85 (31.0%)
Childhood onset chronic condition			
Complex Care	24 (15.3%)	12 (10.3%)	36 (13.1%)
Diabetes Mellitus	32 (20.4%)	18 (15.4%)	50 (18.2%)
EDS/POTS	10 (6.4%)	6 (5.1%)	16 (5.8%)
Gastrointestinal	16 (10.2%)	23 (19.7%)	39 (14.2%)
Sickle Cell	39 (24.8%)	35 (29.9%)	74 (27.0%)
Rheumatological	17 (10.8%)	5 (4.3%)	22 (8.0%)
Other	19 (12.1%)	18 (15.4%)	37 (13.5%)
Type of admission			
Emergency	140 (89.2%)	113 (96.6%)	253 (92.3%)
Urgent or Elective	17 (10.8%)	4 (3.4%)	21 (7.7%)
Admission Source			
Home or non-health care facility	136 (86.6%)	108 (92.3%)	244 (89.1%)
Admission from outpatient facility	8 (5.1%)	1 (0.9%)	9 (3.3%)
Transfer from another hospital	13 (8.3%)	8 (6.8%)	21 (7.7%)
Did patient have ICU stay?			
No ICU Stay	144 (91.7%)	106 (90.6%)	250 (91.2%)
ICU Stay	13 (8.3%)	11 (9.4%)	24 (8.8%)
Discharge disposition			
Home or Self Care	131 (83.4%)	89 (76.1%)	220 (80.3%)
Home Health Service	17 (10.8%)	20 (17.1%)	37 (13.5%)
Self-Directed Discharge	4 (2.5%)	1 (0.9%)	5 (1.8%)
Other	5 (3.2%)	7 (6.0%)	12 (4.4%)
Readmission sample			
No	8 (5.1%)	5 (4.3%)	13 (4.7%)
Yes	149 (94.9%)	112 (95.7%)	261 (95.3%)
*Continuous variables summarized with mean (SD), categorical variables are summarized with frequency (%)			

Patient outcomes

Length of Stay

**Figure 2:**
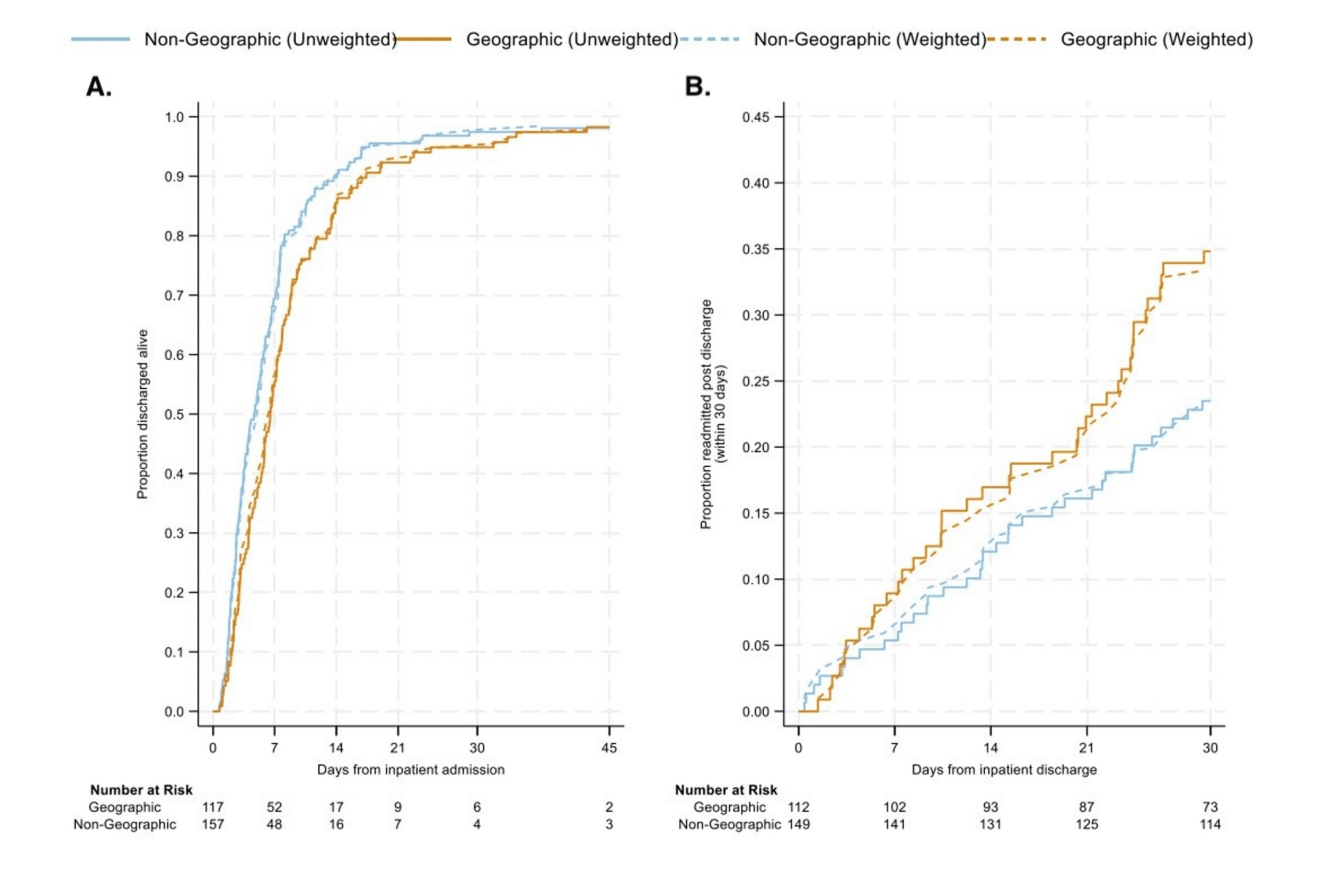
Weighted and unweighted Kaplan-Meier curves for time to discharge (from admission) in days (Panel A) and time to 30-day hospital readmission (from index discharge) in days (Panel B)

In weighted analyses, median length of stay increased from 5.1 days (interquartile range 2.6-7.6) in the non‑geographic period to 6.3 days (IQR: 3.1-10.0) during the geographic period (Table 2). Geographic rounding was associated with a 29% longer time to discharge compared with the non‑geographic period (event time ratio = 1.29, 95% CI: 1.04-1.57) (Table 2). Weighted Kaplan-Meier curves showed that most patients in both periods were discharged within one week (Figure 2A).

**Table 2:** Weighted regression estimated differences between study arms

	Non-Geographic	Geographic	Regression Estimates
Length of hospital stay			
Mean (SD)	7.9 (14.8)	9.1(10.6)	
Median (IQR)	5.1 (2.6,7.6)	6.3 (3.1,10.0)	
Event time ratio (95% CI)			1.29 (1.04,1.57)
Discharged alive? (%)	100.0%	100.0%	
Days to readmission			
Mean (SD)	13.9 (9.3)	15.3 (8.8)	
Median (IQR)	13.4 (6.3,22.3)	15.5 (7.3,24.2)	
Hazard Ratio (95% CI)			1.53 (0.81,2.71)
Readmission within 30 days (%)	23.3%	33.9%	
Risk Ratio (95% CI)			1.45 (0.85,2.41)

Thirty‑Day Hospital Readmission

Thirty‑day readmission rates were numerically higher during geographic rounding. A weighted proportion of 33.9% of patients in the geographic period experienced an unplanned readmission within 30 days, compared with 23.3% in the non-geographic period (risk ratio = 1.45, 95% CI: 0.85-2.41) (Table 2). This difference did not reach statistical significance. Time‑to‑event analyses demonstrated a steady rate of readmission over the follow‑up period in both groups (Figure 2B).

Nursing workforce outcomes

**Figure 3:**
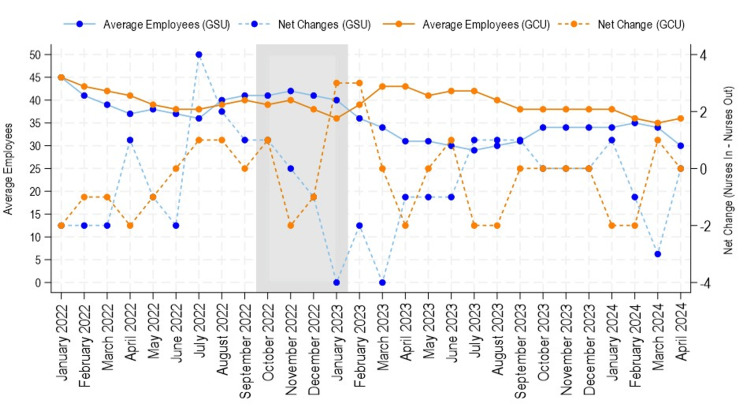
Average nursing staff levels and net monthly change in staff (total ins - total outs) for GSU and GCU between January 2023 and October 2023 GSU: geographic study unit, GCU: geographic comparison unit

Average monthly nursing staffing levels and net monthly staffing changes for the GSU and GCU are shown in Figure 3. During the pilot and geographic periods, the GSU experienced declining staffing levels and sustained net staff losses, beginning during the pilot phase and continuing through mid‑2023. By contrast, staffing levels on the GCU remained relatively stable. Throughout most of the geographic period, average staffing on the GSU remained below that of the GCU.

Nursing workload

**Figure 4:**
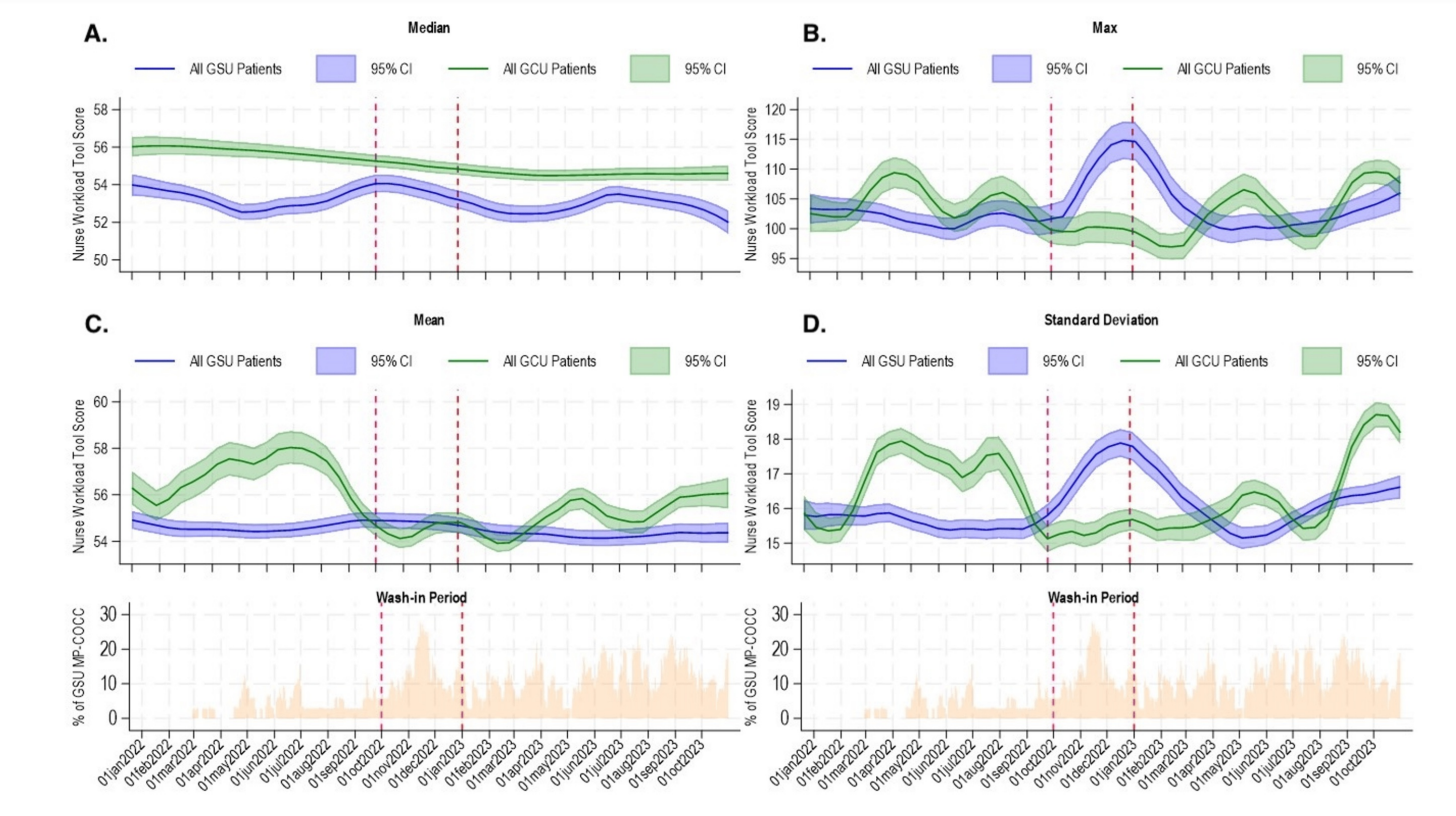
Nurse Workload Total score polynomial smoothing lines for time series of daily summaries with 95% confidence intervals over the study period for GSU and GCU, with total MP-COCC patients on GSU from October 1, 2022 GSU: geographic study unit, GCU: geographic comparison unit, MP-COCC: Internal Medicine-Pediatrics childhood-onset chronic conditions

Daily unit‑level nursing workload scores during the geographic period are presented in Figure 4. Overall workload intensity on the GSU was comparable to the GCU when summarized by daily median (Figure 4A) and mean scores (Figure 4C). However, during the pilot phase, an increasing proportion of MP‑COCC patients on the GSU coincided with greater variability in workload (Figure 4B) and elevated daily maximum workload scores (Figure 4B), in some instances approaching levels typically observed in intensive care settings. These fluctuations were not observed on the GCU and were most pronounced during periods of higher MP‑COCC census.

Nursing response to MP-COCC

**Figure 5:**
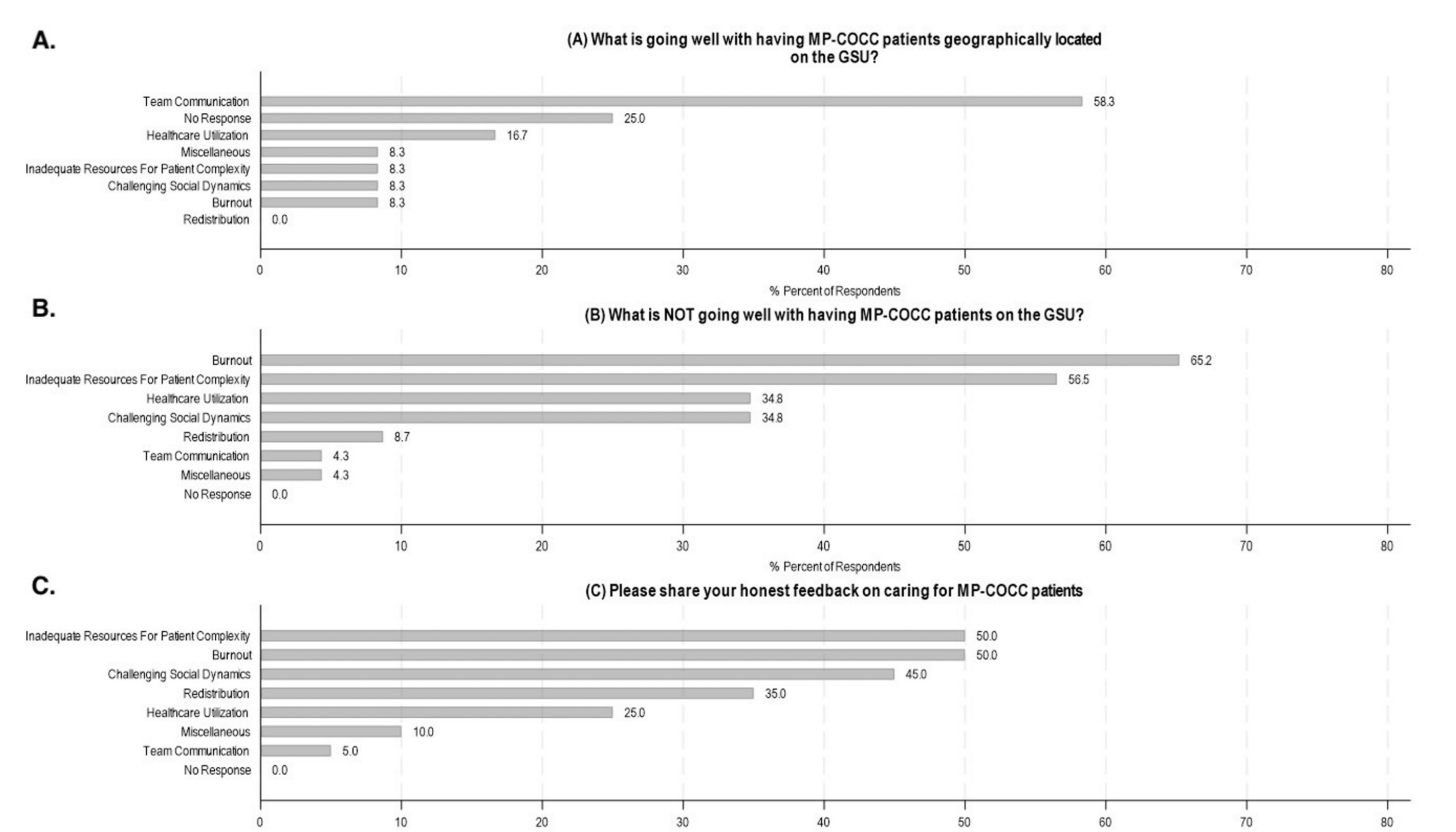
Summary of themes from qualitative answers of floor nurses to three-item open-ended questionnaire (respondent level) GSU: geographic study unit, MP-COCC: Internal Medicine-Pediatrics childhood-onset chronic conditions

**Figure 6:**
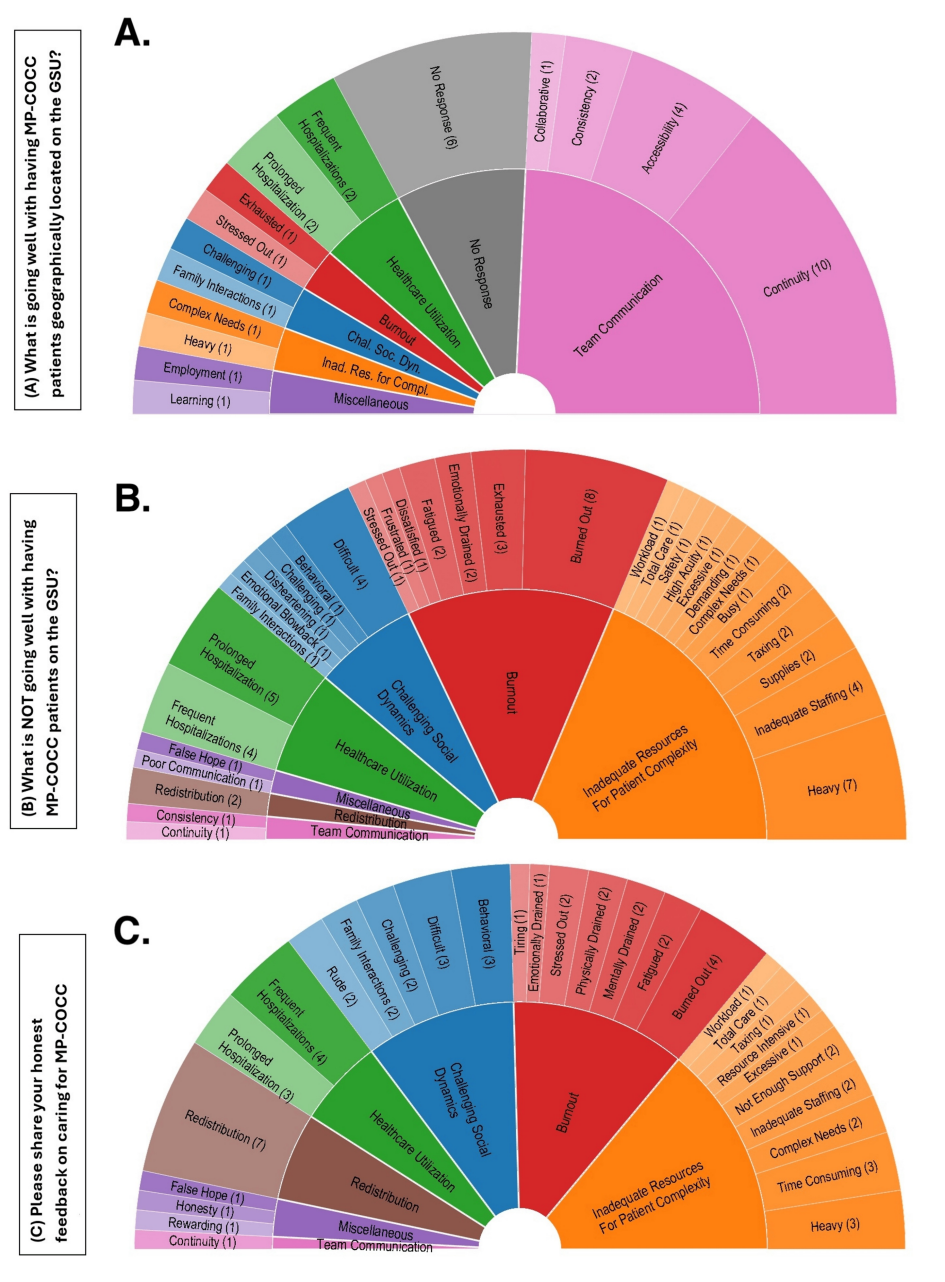
Summary of themes and codes from qualitative answers of floor nurses to three-item open-ended questionnaire (code level) GSU: geographic study unit, MP-COCC: Internal Medicine-Pediatrics childhood-onset chronic conditions

Twenty‑four nurses (47.1% response rate) completed the anonymous free‑response survey. Qualitative analysis identified several consistent themes (Figure 5, Figure 6).

When asked what was going well (Figure 5A, Figure 6A), 25% of participants did not respond. Among responders, the most frequently cited theme was improved team communication (58.3%), with nurses highlighting continuity, familiarity with patients, and improved interdisciplinary collaboration. However, a substantial proportion of respondents reported negative experiences. Themes of burnout (65.2%), inadequate resources to meet patient complexity (56.5%), and challenging social dynamics and perceived inappropriate healthcare utilization (34.8%) were prominent when nurses were asked what was not going well (Figure 5B, Figure 6B).

When invited to provide overall feedback (Figure 5C, Figure 6C), respondents most commonly noted burnout (50%), inadequate staffing or resources for patient complexity (50%), and emotionally demanding social dynamics (45%). Nurses frequently described emotional fatigue, heavy cognitive workload, and frustration related to balancing complex psychosocial needs with existing staffing models.

## Discussion

This is, to our knowledge, the first to evaluate geographic rounding for hospitalized young adults with COCCs, strengthened by a mixed-methods approach that included qualitative analysis of nursing responses to caring for this specific population. In contrast to prior studies demonstrating that geographic rounding combined with MDRs can reduce LOS and readmissions in general medicine populations [5,6], we observed an increase in LOS and no improvement in 30-day readmission rates after implementing geographic rounding on an MP-COCC service. These findings suggest that the benefits observed with geographic rounding in more heterogeneous general medicine populations may not generalize to medically and socially complex populations without additional structural adaptations.

Several factors may explain the observed increase in LOS. Patients on MP-COCC often require processes that may not be accelerated by geographic rounding alone, including extensive care coordination, behavioral support, and transition planning [8]. Additionally, as compared to prior studies that showed improvements in LOS with the implementation of geographic rounding, the majority of patients in this study were discharged home, eliminating the likely benefit to LOS that occurred with the addition of MDR to geographic rounding when it came to arranging placement needs [5,6].

Nursing outcomes provide important contextual insight into these patient‑level results. Despite comparable measured nursing workload between units, the GSU experienced increased nursing attrition and lower staffing levels. Qualitative findings revealed that while communication and familiarity improved, nurses consistently described burnout, challenging social dynamics, and inadequate resources to manage patient complexity. These findings suggest that conventional workload metrics may inadequately capture the cognitive and emotional labor required to care for medically and socially complex young adults, including frequent reassurance, behavioral challenges, and complex family dynamics. Increased variability in workload during periods of higher MP‑COCC census further supports this interpretation.

Implementation challenges likely contributed to these outcomes. Nurses did not receive targeted preparation or training specific to the MP‑COCC population prior to geographic rounding, despite evidence that staff readiness and role clarity are critical to successful geographic models [8,9,15]. Without such preparation, MP‑COCC patients may have been perceived as outliers, disrupting established workflows and contributing to cultural strain on the unit [16]. The heterogeneity of disease processes and psychosocial needs within this group underscores the importance of aligning staffing models, training, and psychosocial supports when implementing geographic interventions. Prior literature emphasizes that geography alone is insufficient; success depends on co‑design with nursing leadership, adequate staffing ratios, and access to ancillary and behavioral health resources [17,18].

This study has limitations. It was conducted at a single academic medical center, limiting generalizability. The sample size was also modest, which may have contributed to large variability in 30-day readmission rates which would have been strengthened by larger sample sizes. The study also excluded the inclusion of patients who were not discharged from the GSU, generating a source of potential selection bias if more complex or prolonged admissions were preferentially housed on the GSU. The retrospective observational design precludes causal inference, and residual confounding may remain despite the use of overlap weighting. Qualitative data were derived from a limited sample of nurses from a narrow set of questions and did not permit follow‑up probing. Nonetheless, the integration of patient outcomes with nursing workforce data and frontline qualitative perspectives strengthens interpretability and highlights critical implementation barriers that may otherwise be obscured in outcome‑only analyses.

## Conclusions

In this mixed‑methods evaluation, geographic rounding for young adults with COCCs was associated with increased length of stay, no improvement in readmission rates, and unintended nursing workforce consequences. These findings indicate that geographic rounding, while effective in some general medicine populations, may be poorly suited to medically and socially complex specialty cohorts without concurrent structural and staffing adaptations.

The implications of this work extend beyond MP‑COCC patients and teams. As hospital medicine continues to sub‑specialize, care delivery interventions should be tailored to the unique needs of both patients and staff at each individual institution. Future implementations should include early and explicit engagement between provider teams, nursing leadership, and hospital administrators to ensure adequate preparation and resource allocation. Geographic models incorporating flexible staffing, multidisciplinary behavioral and social support, and attention to transition readiness may be more effective than geographic rounding alone.

Further research is needed to identify strategies that optimize LOS and patient experience for medically and socially complex populations while safeguarding workforce sustainability. There is no one‑size‑fits‑all approach to streamlining inpatient care; progress will require collaborative, data‑driven efforts grounded in implementation science and informed by frontline experience.
